# Effect of Puffing Behavior on Particle Size Distributions and Respiratory Depositions From Pod-Style Electronic Cigarette, or Vaping, Products

**DOI:** 10.3389/fpubh.2021.750402

**Published:** 2021-12-01

**Authors:** Anand Ranpara, Aleksandr B. Stefaniak, Elizabeth Fernandez, Ryan F. LeBouf

**Affiliations:** Respiratory Health Division, National Institute for Occupational Safety and Health, Morgantown, WV, United States

**Keywords:** e-cigarette, pod-style e-cigarette, JUUL^®^, particle size distributions, respiratory deposition, secondhand exposure estimates

## Abstract

The current fourth generation (“pod-style”) electronic cigarette, or vaping, products (EVPs) heat a liquid (“e-liquid”) contained in a reservoir (“pod”) using a battery-powered coil to deliver aerosol into the lungs. A portion of inhaled EVP aerosol is estimated as exhaled, which can present a potential secondhand exposure risk to bystanders. The effects of modifiable factors using either a prefilled disposable or refillable pod-style EVPs on aerosol particle size distribution (PSD) and its respiratory deposition are poorly understood. In this study, the influence of up to six puff profiles (55-, 65-, and 75-ml puff volumes per 6.5 and 7.5 W EVP power settings) on PSD was evaluated using a popular pod-style EVP (JUUL^®^ brand) and a cascade impactor. JUUL^®^ brand EVPs were used to aerosolize the manufacturers' e-liquids in their disposable pods and laboratory prepared “reference e-liquid” (without flavorings or nicotine) in refillable pods. The modeled dosimetry and calculated aerosol mass median aerodynamic diameters (MMADs) were used to estimate regional respiratory deposition. From these results, exhaled fraction of EVP aerosols was calculated as a surrogate of the secondhand exposure potential. Overall, MMADs did not differ among puff profiles, except for 55- and 75-ml volumes at 7.5 W (*p* < 0.05). For the reference e-liquid, MMADs ranged from 1.02 to 1.23 μm and dosimetry calculations predicted that particles would deposit in the head region (36–41%), in the trachea-bronchial (TB) region (19–21%), and in the pulmonary region (40–43%). For commercial JUUL^®^ e-liquids, MMADs ranged from 0.92 to 1.67 μm and modeling predicted that more particles would deposit in the head region (35–52%) and in the pulmonary region (30–42%). Overall, 30–40% of the particles aerosolized by a pod-style EVP were estimated to deposit in the pulmonary region and 50–70% of the inhaled EVP aerosols could be exhaled; the latter could present an inhalational hazard to bystanders in indoor occupational settings. More research is needed to understand the influence of other modifiable factors on PSD and exposure potential.

## Introduction

Electronic cigarette, or vaping, products (EVPs) heat liquids (“e-liquids”) using a battery-operated coil and deliver the aerosolized particles to the lungs. By 2014, EVPs were the most popular tobacco product among youth in the USA. The evolution of internal design and external appearance of EVPs have occurred with each consequent modification, referred to as “generations” (1–3). The current, fourth generation, EVPs or “pod-mod” or “pod-style” device type includes two parts: a heating coil/e-liquid reservoir assembly and a flow-activated, rechargeable battery. The coil/e-liquid reservoir assembly is referred to as a “pod” and is either a prefilled disposable pod or refillable pod. For consistency in our study, the fourth generation EVP design type is described as pod-style. A pod-style design such as JUUL^®^ brand has been popular for its sleek design, user-friendly function, desirable flavors, and ability to be used for “stealth vaping” (4–14).

Studies have characterized the constituents of JUUL^®^ e-liquids and documented the different proportions of propylene glycol (PG) and vegetable glycerin (VG); other constituents include flavorings, nicotine, and benzoic acid (BA) (15–20). In the presence of BA, nicotine forms a salt, which forms protonated nicotine, rather than free-base nicotine, thereby allowing high levels of nicotine to be inhaled with less irritation or harsh “throat hit” as compared with traditional tobacco cigarettes and earlier generation (first, second, and third) EVP designs (2, 17, 20–23). The literature has conveyed that the presence of nicotinic salts in JUUL^®^ e-liquids maximized nicotine uptake to the blood (8, 17, 21–24). The literature contains reports of the chemical toxicity associated with particles emitted by aerosolizing JUUL^®^ e-liquids (16, 25–28).

Studies have emphasized mass-based particle size distribution (PSD) of EVP aerosols as an influential factor for estimating their regional respiratory deposition during inhalation and exhalation (29–33). Determining the PSD of EVP aerosols has been complex because these liquid droplets can deviate from their innate size depending on various conditions, such as evaporation and hygroscopic growth (34–36). The evaporation of liquid droplets in the EVP aerosol during sampling results in an underestimation of particle size, while hygroscopic growth results in an overestimation of particle size (36). These deviations in measuring PSD, in turn, result in errant predictions of regional depositions in the respiratory tract. Hence, the innate properties of particles should be maintained as intact as possible while measuring to determine an accurate PSD of the emitted EVP aerosols (37, 38). Oldham et al. (37) and Zhao et al. (38) predicted gas and particle phases as a function of the mass of collected aerosols without dilution to keep the physical and chemical components of the aerosol intact.

Previous studies evaluated the effects of multiple interlinked factors such as e-liquid compositions, puffing behavior (puff topography), and EVP electric settings (voltage, power, coil temperature, etc.) on PSD by aerosolizing e-liquid using first, second, and third generation EVPs (39–43). Fuoco et al. (39) found that flavorings from the products studied have a negligible influence on EVP particle emission. Lechasseur et al. (40) discovered that together nicotine and flavorings in 30:70 PG–VG e-liquid did not affect PSD in any of the studied EVP power settings. Robinson et al. (42) presented an empirical correlation model of the dependence of whole aerosol mass emissions as a function of parameters, such as puff flow conditions, device operating power, and e-liquid composition, from the five different types across different EVP generations, including pod-style (JUUL^®^). This group considered the lack of information regarding puff topography for JUUL^®^ as a limitation that needed to be addressed for a better functionality of their model. Vargas-Rivera et al. (43) provided puff topographical data from 21 college-aged (18–24 years) JUUL^®^ users and reported that JUUL^®^ flavored e-liquid usage did not seem to significantly affect puffing behavior, i.e., puff volumes, puff duration, interval, and number of puffs. Hence, EVP users' puffing behavior and PSD may not be affected by the presence of both flavorings and nicotine in PG–VG-based e-liquids. Lechasseur et al. (40) and Son et al. (44) have evaluated multifactor effects on PSD and lung deposition, but these studies did not include pod-style EVPs. Therefore, it is critical to assess the impact of multiple factor variations on measuring the PSD of pod-style EVPs to better understand their role in aerosol deposition in the respiratory tract.

Considering the extensive use of JUUL^®^ pods and the availability of other refillable pods (identical or not with JUUL^®^ brand), the impact of influential factors affecting PSD and ultimately respiratory deposition as a significant knowledge gap must be addressed. Through this study, we offer insights into inhaled respiratory depositions and exhaled potential exposure fraction of aerosols impacted by puff volumes and power settings using both types of pod-style products: refillable or prefilled. We also assess lung deposition and, thereby potential secondhand exposure fraction, as a function of mass-based PSD of a JUUL^®^-like laboratory prepared reference e-liquid using refillable pods compared with commercially available prefilled JUUL^®^ pods impacted by various puff behavior patterns. Stefaniak et al. (16) conveyed the mismatch between laboratory prepared study materials and widely used market EVPs, limiting the generalizability of research applications in real-world scenarios. By including both, we captured the standardized approach of controlling the preparation of e-liquids for testing and the generalizability of testing commercial e-liquids contained in the sealed pod-style devices, which more closely matches real-world conditions.

## Materials and Methods

### Reference e-Liquid in Refillable Pods

As noted, the constituents of JUUL^®^ e-liquids include PG–VG, acids, ethanol, flavorings, nicotine, and water (15, 16, 19, 20). Talih et al. (17) reported that the ratio of PG–VG was 30:70–27:73 in both liquid and aerosol for the JUUL^®^ products sold in the USA (17, 18). Mallock et al. (7) reported that the PG–VG ratio of JUUL^®^ e-liquid was 25:60 and the concentration of BA was ~9.4 mg/ml. Reilly et al. (26) evaluated toxicant emission considering 30:70 PG–VG to mimic JUUL^®^ e-liquids. As flavor and nicotine together do not affect puff topography and PSD, they were not included in the preparation of the reference e-liquid (40, 43). Hence, we prepared an e-liquid, known as the “reference e-liquid,” which mimics the makeup of JUUL^®^ using a composition of 25:73 PG (ACS grade, CAS# 57-55-6, Fisher, Hampton, NH, USA)/VG (certified ACS grade, CAS# 56-81-5, Fisher, Hampton, NH, USA) or PG–VG, and 1 part each of 200 proof ethanol (ACS/USP grade, CAS# 64-17-5, Pharmaco-Aaper, Brookfield, CT, USA) and 18 mΩ water with BA concentration at 9.4 mg/ml. Preparations were made using a Mettler Toledo XS 250 dual range microbalance (Mettler-Toledo LLC, Columbus, OH, USA). Although JUUL^®^ does not explicitly list its constituent concentrations, the reference e-liquid constituent concentrations were selected to mimic the suspected composition of commercially available JUUL^®^ pods. Approximately 0.7 ± 0.03 ml of the reference e-liquid was placed into refillable pods (Blankz! brand pods), which simulate the volume, appearance, and style of JUUL^®^ brand pods. The density (1.13 ± 0.02 g/ml) of the reference e-liquid was determined by measuring the gravimetric mass of the e-liquid at a volume of 100 μl in triplicate. The volume of the reference e-liquid in the pod was calculated by dividing the pre- and post-mass difference by the density.

### JUUL^®^ e-Liquid in Disposable Pods (JUUL Labs, Inc., San Francisco, CA, USA)

We studied 12 commercially available JUUL^®^ pod types of varying flavors and nicotine content, which were currently or previously commercially available. In November 2019, JUUL^®^ voluntarily stopped the sale of all but Classic Menthol-, Classic Tobacco-, and Virginia Tobacco- flavored e-liquids. The US Food and Drug Administration (FDA) announced on January 2, 2020, its final enforcement policy for removing prefilled, flavored e-liquid cartridge-based products from the US market (except Menthol and Tobacco flavors) (45). Moreover, consumers can still purchase concentrated humectants or flavorings to dilute and mix their own desired flavored e-liquid, and then fill their pods at home (46). Three independent measurements were conducted to calculate the density (g/ml) of JUUL^®^ e-liquids by measuring the gravimetric mass of the JUUL e-liquid at a fixed deliverable volume. Details of the commercial availability of JUUL^®^ pod e-liquids used in this study are described in Table 1.

**Table 1:** Details of 12 JUUL^®^ pod types used in this study.

**JUUL^®^ pod type**	**Nicotine %**	**Market availability**
Menthol	3 and 5	Available since 2019
Virginia Tobacco	3 and 5	Available since 2019
Classic Tobacco	3 and 5	Discontinued May 8, 2020
Mint	3 and 5	Discontinued November 7, 2019
Crème Brulee	5	Discontinued October 17, 2019
Fruit Medley	5	Discontinued October 17, 2019
Mango	5	Discontinued October 17, 2019
Classic Menthol	5	Limited Edition available in 2019

### Experimental Design

An automated e-cigarette aerosol generator (ECAG+, e^~^Aerosols LLC, Central Valley, NY, USA) was programmed to aerosolize the reference e-liquid and JUUL^®^ e-liquids. The ECAG+ works using positive pressure to aerosolize e-liquids. Prior to conducting trials, both JUUL^®^ pods and the reference e-liquid pod were puffed 10 times to ensure that the coil was heated at the calibrated puff topography. Puff volumes were calibrated using a soap-bubble flow meter (Borgwaldt KC GmbH, Hamburg, Germany) as a primary volumetric flow calibration device.

JUUL^®^, as a puff flow-activated, low-powered EVP, has been described by the manufacturer as operating at 6–8 W of power settings. However, the user of a JUUL device has no control over the power setting at which e-liquid gets aerosolized between 6 and 8 W. Talih et al. (17) reported the peak operating temperature to ~215°C while conducting their study with JUUL^®^ EVPs at the maximum power (i.e., 8.1 W). In this study, reference e-liquid was aerosolized by heating it at 6.5 and 7.5 W [presumably to a temperature <215°C based on the report by Talih et al. (17)]; at three puff volumes, 55, 65, and 75 ml; and with 4-s puff durations that were 30 s apart, which is comparable to previous studies (42, 43, 47–49) and in agreement with the modified CORESTA method (50). Puff flow rates at the three tested puff volumes: 55, 65, and 75 ml with a 4-s puff duration were 0.8, 1.0, and 1.1 LPM, respectively. For commercially available JUUL^®^ pods, one puff topography (65 ml at 7.5 W) was used to aerosolize the e-liquid. This profile was chosen after analyzing the results of the puff profiles for the reference e-liquid.

In our temperature- and humidity-controlled laboratory study, EVP aerosols were directly sampled with minimal dilution into a low-flow cascade impactor to keep the native physical properties of the aerosol intact as detailed in previous studies (37, 38, 51). MiniMOUDI™ (MSP Corporation, Shoreview, MN, USA) was used to size fractionate EVP aerosols (size range: 0.056–10 μm) at a sampling flow rate of 2.0 LPM. The mass of aerosols deposited on each impactor stage was measured on a 37-mm aluminum filter using a Mettler Toledo XS 205 dual range microbalance with a mass resolution of 0.01 mg (Mettler-Toledo LLC, Columbus, OH, USA). Five trials with five puffs per trial were conducted for each JUUL^®^ pod type, and three trials with three puffs per trial were conducted using the reference e-liquid in a refillable pod. Considering intra-device variability with the aerosol generation with JUUL^®^ pods reported by Mallock et al. (7), we conducted five trials with five puffs per trial using JUUL^®^ pods compared to three trials with three puffs per trial using the reference e-liquid in a refillable pod.

**Figure 1:**
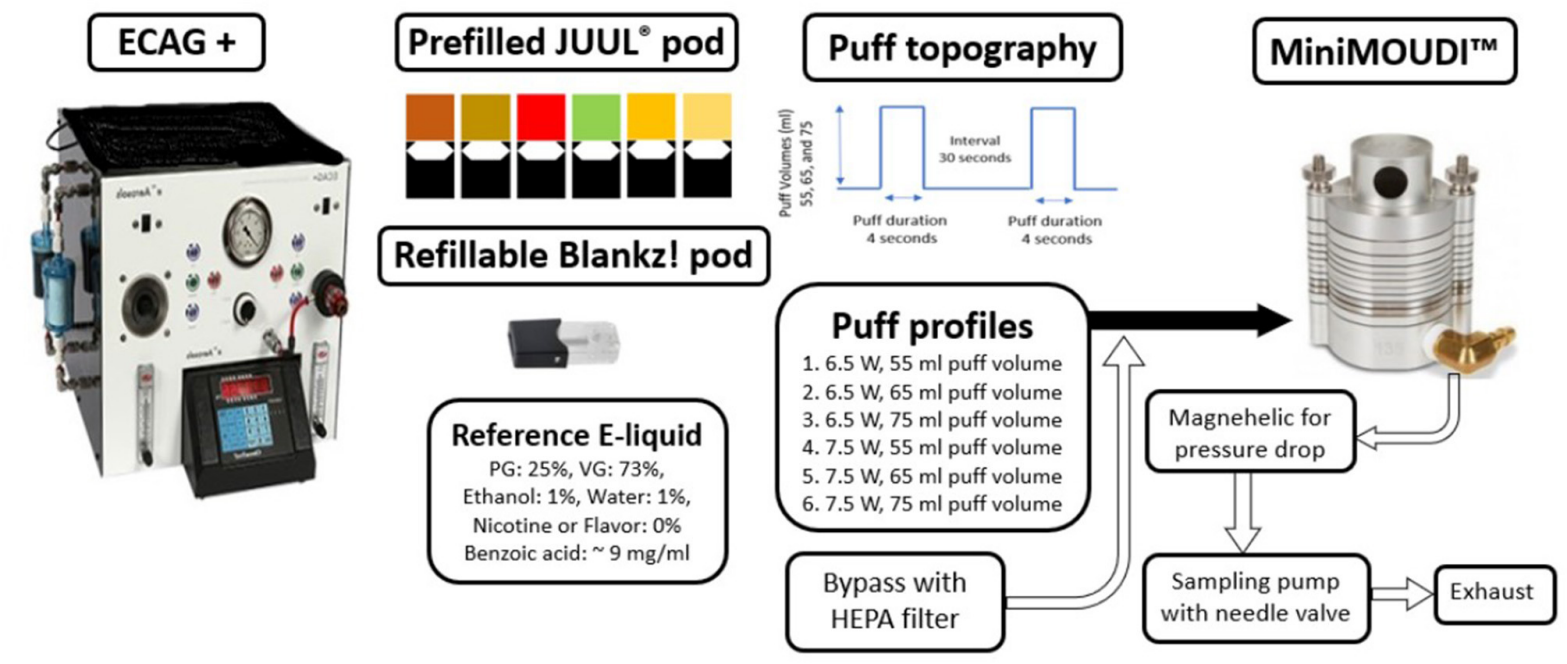
Schematic of the experimental setup. ECAG+, Electronic cigarette aerosol generator plus. HEPA, High efficiency particular air.

Figure 1 shows a schematic of the experimental setup. The ECAG+ powered the pod and forced the emitted EVP aerosols with the established puff topography into the MiniMOUDI™. While the ECAG+ was operating, there was a puff flow rate of 0.8, 1.0, and 1.1 LPM at the puff volumes of 55, 65, and 75 ml, respectively. The rest of the sampling flow rate of 2.0 LPM entered from a bypass air flow that passes through a high efficiency particulate air filter (HEPA; Whatman^®^ Schleicher & Schuell; Stockbridge, GA, USA) to allow uninterrupted flow to the MiniMOUDI™ and to alleviate pressure drops because of differences between the EVP aerosol puff flow rate and sampling flow rate. During the puff delay, the impactor sampled 2.0 LPM from bypass air, which did not result in any mass loading on the aluminum filters. To minimize aerosol loss, the pods were connected to an inlet of the MiniMOUDI™ using flexible, black conductive silicone tubing with an inside diameter of 0.5 cm and a length of 70 cm.

#### Evaporative Aerosol Mass Loss During Sampling for Reference e-Liquid

Depending on sampling and experimental parameters, the evaporation and hygroscopic growth of liquid droplets in the EVP aerosol result in a biased estimation of PSD (36), and eventually, in a biased estimation of lung deposition. For similar types of EVPs, Oldham et al. (37) reported a larger particle size while sampling at low dilution, whereas Mikheev et al. (41) noted a reduced size of particles because of evaporation at elevated flow rates of more than 25 ml/s. Flow rates used in this study correspond to 14–19 ml/s. We measured mass loss in the collected EVP aerosol at each puff volume of 7.5 W to determine whether it affected mass median aerodynamic diameters (MMADs) and associated respiratory deposition using the reference e-liquid. *Post-hoc*, we only compared evaporative mass loss for 55- and 75-ml puff profiles at 7.5 W based on a significant influence of these parameters on MMADs (see Section Results). Mass loss trials were conducted by collecting reference e-liquid aerosols using an impactor in the same manner as described previously. After collection, the filter stages were returned to an impactor and clean air was sampled at a flow rate of 2.0 LPM, at increasing times (1, 5, and 15 min). Deposited mass on each filter stage was measured after loading the EVP aerosol followed by clean air at increasing time intervals. Linear regression models were used to calculate the mass deposited for each size bin at time zero, which was the mass used to calculate an adjusted MMAD. Non-adjusted MMADs were compared to the adjusted MMADs for each puff profile.

### Data Analysis

Data were log transformed and managed using JMP 15.1.0 (SAS Institute, Inc., Cary, NC, USA). Deposited mass (in mg) of EVP aerosols for each impactor stage of the MiniMOUDI™ was calculated by measuring aluminum substrate before (pre-) and after (post-) sampling. To demonstrate mass-based PSD, MMAD and geometric SD (GSD) were calculated by including each cutoff size for the impactor stages of the MiniMOUDI™ using a probit model (52). Data acquisition of trials (*n* = 3 or *n* = 5) was done at least in triplicate, and significant differences (*p* < 0.05) between MMADs were determined using an one-way ANOVA and Tukey's HSD multiple comparisons.

#### Respiratory Deposition

The fraction of particles estimated to deposit in the respiratory tract was calculated using the Multiple Path Particle Dosimetry (MPPD), version 3.04 (ARA, Albuquerque, NM, USA). Regional depositions were reported as mass deposited in the head, trachea-bronchial (TB), and pulmonary regions using a default adult human symmetric model as an oronasal mouth breather (the Yeh–Schum model) in an upright position. The Yeh–Schum model uses a symmetric tree for the whole lung, as given by Yeh and Schum (53), and the estimated results correspond with the results from realistic lung structures (54). Regions from the mouth, nose, larynx, pharynx to trachea (generation 0) are considered as head. Regions from the trachea (generation 0) to the bronchioles (generation 16) are considered as TB. Regions beyond terminal bronchioles (from generations 16–23) are considered as pulmonary. Total respiratory deposition fractions were calculated by summing the regional deposition fractions. Regional deposition (%) was calculated by the fraction of aerosols deposited in each region (head, TB, and pulmonary) divided by the total respiratory deposition. Statistical differences between the respective regional depositions of the reference e-liquid and JUUL^®^ e-liquids at each studied puff profile were tested using an one-way ANOVA at *p* < 0.05. Based on the total respiratory deposition, the exhaled aerosol fraction was estimated using Equation 1.


(1) $$\begin{array}{l} \begin{array}{c} Exhaled\;aerosol\;fraction \end{array}\\ \begin{array}{c} \begin{array}{r} \begin{array}{c} \;=1-Total\;respiratory\;deposition\;fraction \end{array} \end{array} \end{array} \end{array}$$

The Yeh–Schum single path model considered the whole lung as a symmetric tree; therefore, respective regional depositions are the average values for each generation in the head, TB, and pulmonary regions (53). Unlike regional deposition, the Yeh–Schum lobular deposition pattern characterized the segmental bronchi within each lobe as a single symmetric path to report mass deposited in each of the five lobes of the lungs: right upper (RU), right middle (RM), right lower (RL), left upper (LU), and left lower (LL) (53). The total lobular deposition includes depositions in the TB and pulmonary regions of each lung lobe but not the initial airways as they do not belong to any lobe. Default parameters for the Yeh–Schum model were as follows: forced residual capacity = 3,300 ml, upper respiratory tract volume = 50 ml, breaths per minute (bpm) = 12, and tidal volume = 625 ml.

## Results

### PSD and Respiratory Depositions for Reference e-Liquid

Of the six puff profiles studied, larger MMADs and wider GSDs were reported as puff volumes increased from 55 to 65 ml when heating the reference e-liquid at 7.5 W compared with 6.5 W (Table 2). We measured a statistical difference between MMADs for 55- (MMAD 1.23 μm) and 75-ml (MMAD 1.02 μm) puff volumes when heated at 7.5 W. The results for evaporative mass loss adjusted trials for these two puff profiles at 7.5 W are also reported in Table 2. This observation suggested a significant impact of puff volume (55 and 75 ml) on the PSD of the reference e-liquid aerosolized at 7.5 W. Irrespective of power settings, a 75-ml puff volume resulted in a narrower PSD (GSD 1.45–1.56) compared with other puff volumes (GSD 1.60–1.70). The largest average MMAD ± SD (1.23 ± 0.02 μm) and GSD (1.70) resulted when heating the reference e-liquid at 7.5 W at a 55-ml puff volume across all the tested puff profiles. The smallest average MMAD ± SD (1.02 ± 0.08 μm) and GSD (1.45) for all the tested puff profiles were reported on heating the reference e-liquid at 7.5 W at a 75-ml puff volume. We did not notice a statistical significance among MMADs and mass loss adjusted MMADs at these two puff volumes: 55 (MMAD 1.23 vs. 1.23 μm) and 75 ml (MMAD 1.02 vs. 1.00 μm) at 7.5 W. Because evaporative mass loss did not affect MMADs at the puff flow rates of 14 and 19 ml/s (55 and 75 ml volumes over 4 s), no loss correction was made to the rest of the trial mass data.

**Table 2:** Particle size distribution (PSD), regional respiratory deposition, and exhaled aerosol for the reference e-liquid at different puff profiles with three puffs (*n* = 3 per profile).

**Puff profiles**	**Power (W)**	**Puff volume (ml)**	**Average MMAD (μm) ± SD**	**Average GSD**	**Head (%)**	**TB (%)**	**Pulmonary (%)**	**Total respiratory (%)**	**Exhaled aerosol (%)**
1	6.5	55	1.10 ± 0.01^A, B^	1.60	38	20	42	32	68
2		65	1.11 ± 0.03^A, B^	1.60	38	20	42	31	69
3		75	1.10 ± 0.07^A, B^	1.56	38	20	42	31	69
4	7.5	55	1.23 ± 0.02^A^	1.70	41	19	40	36	64
4*		55	1.23 ± 0.03^A^	1.70	41	19	40	36	64
5		65	1.13 ± 0.06^A, B^	1.63	39	20	41	32	68
5*		65	1.11 ± 0.08^A, B^	1.58	39	20	41	32	68
6		75	1.02 ± 0.08^B^	1.45	36	21	43	28	72
6*		75	1.00 ± 0.07^B^	1.45	36	21	43	28	72

* *Indicates puff profiles evaluated for evaporative mass loss*.

*MMAD, mass median aerodynamic diameter; SD, standard deviation; GSD, geometric SD; TB, trachea-bronchial region*.

*MMAD, if not connected by the same letter (A or B), are significantly different at p < 0.05. Resistance (Ohm: Ω) of the reference e-liquid containing Blankz! pod: Average ± SD (1.87Ω ± 0.02 Ω) with relative SD 1.30%*.

Particle size distributions did not differ among the puff volumes 55, 65, and 75 ml when the reference e-liquid was aerosolized at 6.5 W (Table 2). Furthermore, we did not observe statistically significant differences between the MMADs of the reference e-liquid aerosolized at a 65-ml puff volume when compared with the 55- and 75-ml puff volumes, irrespective of power settings. MMAD and GSD results for individual trials, including mass loss trials for all e-liquids, are presented in Supplementary Table 1.

Based on MMADs and GSDs, the dosimetry analysis estimated more mass fractional deposition in the pulmonary region (40–43%) than in the head (36–41%) and TB regions (19–21%). Generally, across all the studied puff profiles (except the 55-ml puff volume at 7.5 W), the highest regional deposition was estimated to be at the pulmonary region. Deposition in the pulmonary region accounted for ~40% or more of the total deposited aerosol. Dosimetry analysis revealed the highest pulmonary (43%) and TB (21%) deposition at a 75-ml puff volume at 7.5 W. At this puff profile, smaller particle sizes with an average MMAD ± SD (1.02 ± 0.08 μm) and a tighter aerosol size distribution with lesser GSD (1.45) resulted in higher mass deposition in pulmonary and TB regions compared with the head. A wider PSD with a greater GSD (1.70) and a higher MMAD (1.23 μm) was noticed with a higher deposition in the head compared with the other regions of 55-ml puff volume at 7.5 W. In the same puff profile, the only exception resulted in the highest deposition in the head region (41%), based on the average MMAD ± SD (1.23 ± 0.02 μm) and GSD (1.70) at the 65- and 75-ml puff volumes. Although regional respiratory deposition was comparable between pulmonary (40%) and head regions (41%) for the puff profile of a 55-ml puff volume at 7.5 W. As expected, a larger MMAD ± SD (1.23 ± 0.02 μm) and GSD (1.70) from a 55-ml puff volume at 7.5 W resulted in a higher deposition in the head.

Comparing 55- and 75-ml puff volumes between the power settings, a wider range in the estimated total respiratory deposition was noticed at 7.5 W (28–36%) compared to 6.5 W (31–32%). Generally, all the tested puff profiles resulted in 19–21% deposition in the TB region. Puff profiles with smaller MMADs resulted in a higher pulmonary deposition, lower total respiratory deposition, and higher percentage of exhaled aerosol.

Inhaled total respiratory deposition and the percentage of exhaled EVP aerosols were inversely related. Hence, the lower the total deposition, the higher the percentage of exhaled EVP aerosols. Depending on MMAD and GSD, the highest total respiratory deposition (36%) and thereby the lowest percentage of exhaled EVP aerosols (64%) were from a puff profile of 55-ml puff volume at 7.5 W. Inversely, the lowest total respiratory deposition (28%) and thereby the highest percentage of exhaled EVP aerosols (72%) resulted from a puff profile of 75-ml puff volume at 7.5 W. Like MMAD and GSD values, the remaining puff profiles, other than those with 55 and 75 ml of puff volumes at 7.5 W, have similar regional depositions (head: 38–39%, TB: 20%, and pulmonary: 41–42%), total respiratory depositions (31–32%), and the percentage of exhaled EVP aerosols (68–69%). Operating at a higher power setting of 7.5 W was an influential factor affecting PSD; and 65 ml of puff volume, at which the PSD did not result into a statistical difference across other puff profiles, was considered to aerosolize the prefilled JUUL^®^ pods for evaluating PSD, and thus, their *vis-à-vis* comparisons for respiratory depositions.

### PSD and Respiratory Depositions for JUUL^®^ e-Liquids

Table 3 shows comparisons of e-liquid densities, MMAD, and GSD (for *n* = 5) of the prefilled commercial JUUL^®^ pods with 3 and 5% nicotine strengths aerosolized with a 65-ml puff volume emitted at 7.5 W. Overall, for all the flavors, higher e-liquid density (g/ml) resulted for the 3% nicotine strength compared with the 5%, except for the flavor Classic Tobacco (3%: 1.17 vs. 5%: 1.26). Of 12 JUUL^®^ pod types, the largest averages of MMAD ± SD (1.67 ± 0.53 μm) and GSD (2.75) were reported with the Fruit Medley flavor with a 5% nicotine strength (Fruit Medley 5%). The smallest averages of MMAD and SD (0.92 ± 0.19 μm) and GSD (1.69) were for Menthol with a 5% nicotine strength across all the studied JUUL^®^ pod types. At 5% nicotine, significant differences (at *p* < 0.05) were observed among MMADs for Menthol (0.92 μm) and the three flavor types: Fruit Medley (1.67 μm; *p* = 0.01), Classic Menthol (1.59 μm; *p* = 0.02), and Mango (1.55 μm; *p* = 0.03). We detected no significant differences among MMADs for the rest of the JUUL^®^ pod flavors at each nicotine strength. When compared with Menthol at a 5% nicotine strength (Menthol 5%: GSD 1.69), the rest of the JUUL^®^ pod flavors had wider PSDs based on the GSD values (2.27–2.75).

**Table 3:** PSD, regional respiratory deposition, the percentage of exhaled electronic cigarette, or vaping, products (EVPs) aerosols, and statistical comparisons of JUUL^®^ pod types (*n* = 5 puffs per pod type) and the reference e-liquid at the same puff profile (65-ml puff volume at 7.5 W).

**JUUL^®^ pod type**	**Nicotine %**	**Density of e-liquid (g/ml)**	**Average MMAD (μm) ± SD**	**GSD**	**Head (%)**	**TB (%)**	**Pulmonary (%)**	**Total respiratory (%)**	**Exhaled aerosol (%)**
Virginia Tobacco	3	1.10	1.49 ± 0.30^A, B^	2.50	49	18	33	45	55
Virginia Tobacco	5	1.01	1.20 ± 0.45^A, B^	2.27	43	21	37	36	64
Menthol	3	1.42	1.11 ± 0.11^A, B^	2.45	43	20	37	39	61
Menthol	5	1.27	0.92 ± 0.19^B^	1.69	35	23	42	28	72
Classic Tobacco	3	1.17	1.47 ± 0.34^A, B^	2.54	49	18	33	44	56
Classic Tobacco	5	1.26	1.40 ± 0.14^A, B^	2.57	48	18	34	45	55
Mint	3	1.07	1.21 ± 0.20^A, B^	2.46	45	19	36	40	60
Mint	5	1.06	1.41 ± 0.25^A, B^	2.64	49	18	33	45	55
Crème Brulee	5	1.26	1.31 ± 0.29^A, B^	2.71	48	18	33	42	58
Fruit Medley	5	1.02	1.67 ± 0.53^A^	2.75	52	18	30	47	53
Mango	5	1.03	1.55 ± 0.11^A^	2.65	50	18	32	48	52
Classic Menthol	5	1.11	1.59 ± 0.20^A^	2.57	50	18	32	47	53
Reference	0	1.13	1.13 ± 0.06^A, B^	1.63	39	20	41	32	68

*MMAD, mass median aerodynamic diameter; SD, standard deviation; GSD, geometric SD; TB, trachea-bronchial region*.

*JUUL^®^ pod types and the reference e-liquid not connected by the same letter (A or B) had significantly different MMADs at p < 0.05. Resistance (Ω) for JUUL^®^ pod types: Average ± SD (Ω: 1.81 ± 0.03) with relative SD 1.87%*.

Table 3 also shows comparisons between MMADs of 3% and 5% nicotine for the same JUUL^®^ flavor types; there was no significant difference between the average MMAD of 3% and 5% nicotine for JUUL^®^ flavor types: Classic Tobacco (*p* = 0.65), Virginia Tobacco (*p* = 0.27), Mint (*p* = 0.20), and Menthol (*p* = 0.08). Other than Mint, all JUUL^®^ e-liquid flavors with 5% nicotine strength had smaller MMADs compared with that of the 3% nicotine strength; for Menthol flavor, the average MMAD and GSD at 5% nicotine (0.92 and 1.69 μm) were less than those at 3% nicotine (1.11 and 2.45 μm). A similar observation resulted for the Virginia Tobacco flavor when comparing average MMAD and GSD between 5% nicotine (1.20 and 2.27 μm) and 3% nicotine (1.49 and 2.50 μm). Likewise, Classic Tobacco resulted in a smaller average MMAD and GSD (1.40 and 2.57 μm) at 5% nicotine when compared to 3% nicotine (1.47 and 2.54 μm). However, with Mint flavor type, this trend reversed, and 3% nicotine resulted in a smaller average MMAD and GSD (1.21 and 2.46 μm) than the average MMAD and GSD at 5% nicotine (1.41 and 2.64 μm).

Based on MMAD and GSD, the highest regional respiratory deposition was generally in the head (35–52%), compared with pulmonary (30–42%) and TB regions (18–23%). With the smallest MMAD and GSD, Menthol 5% (0.92 and 1.69 μm) had the highest pulmonary (42%) and TB (23%) depositions and the lowest head (35%) depositions. A smaller particle size with a smaller average MMAD and a tighter PSD with a smaller GSD resulted in higher regional depositions in the pulmonary and TB region when compared with the head. A wider PSD with a larger GSD and a higher MMAD were observed with a higher deposition in the head region compared with the other regions. The dosimetry analysis of the Fruit Medley pod type resulted in the lowest pulmonary (30%) deposition and the highest head (52%) deposition because of a wider PSD and the highest MMAD (1.67 and 2.75 μm). According to the dosimetry analysis, the range of total respiratory deposition for the studied JUUL^®^ pods was estimated to be between 28% (Menthol 5%) and 48% (Mango 5%) with an average of 42 ± 6%. This assessment suggests that ~52–72% of exhaled aerosol with an average of 68% could serve as an aerosol available for secondhand exposure to bystanders.

The average measured density of the reference e-liquid at 1.13 ± 0.02 g/ml was comparable to all the studied JUUL^®^ brand e-liquids at 1.15 ± 0.13 g/ml. None of the studied JUUL^®^ pods were found to have a statistically significant difference from the reference e-liquid and filled in blank pods, when aerosolized at the same puff profile (65-ml puff volume at 7.5 W). Depending on PSD, most of the JUUL^®^ pods with the exception of Menthol with 5% nicotine resulted in a higher regional deposition in the head (43–52%) and a higher total respiratory deposition (36–48%), but a lower percentage of exhaled aerosol (52–64%) when compared with the reference e-liquid, respectively, 39% (head), 32% (total), and 68% (exhaled). These higher regional depositions in the head were attributed to higher MMADs and higher GSD values for JUUL^®^ pods compared to the reference e-liquid (GSD 1.63) with the exception of Menthol at 5% nicotine (GSD 1.69 and the head deposition percentage of 35%). The pulmonary deposition of the reference e-liquid (41%) and Menthol 5% (42%) was similar at the same puff profile (65-ml puff volume at 7.5 W). However, Menthol 5% resulted in lesser total respiratory deposition (28 vs. 32%) and thereby a higher percentage of exhaled EVP aerosols (72 vs. 68%) compared with the reference e-liquid.

The average measured density of the reference e-liquid at 1.13 ± 0.02 g/ml was comparable to all the studied JUUL^®^ brand e-liquids at 1.15 ± 0.13 g/ml. None of the studied JUUL^®^ pods were found to have a statistically significant difference from the reference e-liquid and filled in blank pods, when aerosolized at the same puff profile (65-ml puff volume at 7.5 W). Depending on PSD, most of the JUUL^®^ pods with the exception of Menthol with 5% nicotine resulted in a higher regional deposition in the head (43–52%) and a higher total respiratory deposition (36–48%), but a lower percentage of exhaled aerosol (52–64%) when compared with the reference e-liquid, respectively, 39% (head), 32% (total), and 68% (exhaled). These higher regional depositions in the head were attributed to higher MMADs and higher GSD values for JUUL^®^ pods compared to the reference e-liquid (GSD 1.63) with the exception of Menthol 5% (GSD 1.69 and the head deposition percentage of 35%). The pulmonary deposition of the reference e-liquid (41%) and Menthol 5% (42%) was similar at the same puff profile (65-ml puff volume at 7.5 W). However, for inversely related dosimetry attributes, Menthol 5% resulted in lesser total respiratory deposition (28 vs. 32%) and thereby a higher percentage of exhaled EVP aerosols (72 vs. 68%) compared with the reference e-liquid.

### Lobular Depositions

**Figure 2:**
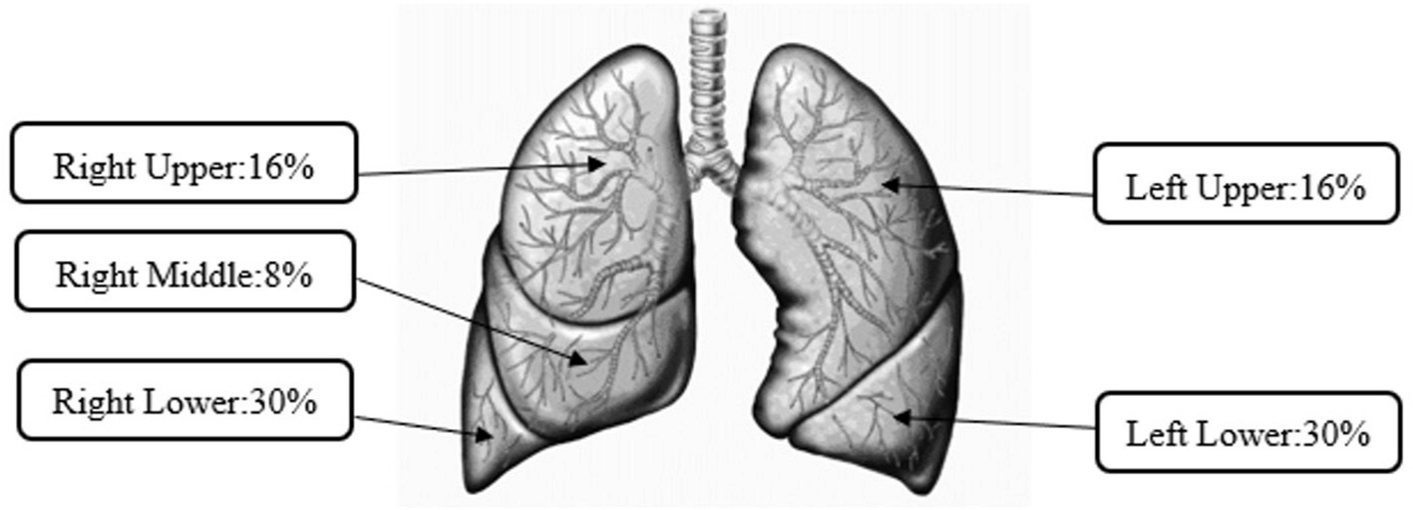
Average lobular deposition of aerosols from pod-style electronic cigarette, or vaping, products (EVPs).

Figure 2 represents lobular aerosol deposition of all the studied puff profiles for the reference e-liquid as well as commercially available prefilled JUUL^®^ pods, which were not significantly different from each other. A slightly higher percentage (8%) of the mass deposited in right-sided lung lobes (sum of RU, RM, and RL = 54%) than left-sided lung lobes (sum of LU and LL = 46%). Manigrasso et al. (55) presented right lung lobes as the sites where the effects of EVP aerosols occur more frequently. For all the studied puff profiles for the reference e-liquid, the MMAD ranged from 1.02 to 1.23 μm and for all commercially available prefilled JUUL^®^ pods, the MMAD ranged from 0.92 to 1.67 μm. The highest percentages of lobular deposition of the emitted aerosols were predicted in the lower lobes (right 30% ± 0.2% and left 30% ± 0.2%) compared with other lobes of the lungs (RU 16% ± 0.2%, RM 8% ± 0.1%, and LU 16% ± 0.2%).

## Discussion

Electronic cigarette, or vaping, products are currently the most popular tobacco product among youth in the USA (56–58). Implications of EVP use also extend to workplaces. Romberg et al. (59) concluded that vaping and vaping exposures are common in US workplaces. Employees, especially non-users, reported reduced productivity because of workplace vaping. Previous studies also purported potential harmful health effects to bystanders because of an exposure to toxic chemical and aerosol emissions from using the fourth generation EVPs, which also negatively affect indoor air quality (59, 60). As such, understanding the emission characteristics of EVPs, specifically PSD, is critical to evaluate aerosol dosimetry for users and bystanders.

The choice of EVP and e-liquid to study aerosol PSD is complex. The current study focused on pod-mod or pod-style EVPs as these fourth generation devices are the most current designs on the marker. The choice of e-liquid includes reference or standardized e-liquids or commercially available products and each has its own unique advantages and disadvantages. Several investigators have created reference or standardized e-liquids for testing earlier generation EVPs (15, 61). Reilly et al. (26) found no differences in free radicals and carbonyl yields when a commercial JUUL^®^ e-liquid was replaced with a laboratory prepared e-liquid with a 30:70 PG–VG mixture that mimicked the PG:VG ratio in the JUUL^®^ e-liquid. This observation was important because commercial brands such as JUUL^®^ do not reveal the formulation of their e-liquids, and therefore it can be difficult to interpret study results when conclusions are made in circumstances where e-liquid composition was not controlled. Building on the results of Reilly et al. (26), we incorporated both attributes of reference and commercial e-liquids using pod-style EVPs: (1) laboratory-prepared reference e-liquid that mimics commercially available JUUL^®^ e-liquid and (2) commercially available JUUL^®^ e-liquids. Our approach had helped to better understand the factors that influence the PSD of these e-liquids measured under the conditions that maintain their native state and provided comparisons of respiratory deposition estimates for users and bystanders.

### Reference e-Liquids

Our study observed a significant influence of puff volumes (55 and 75 ml) on MMADs when heated at a higher power setting (7.5 vs. 6.5 W) (*p* < 0.05). Modifiable factors of the puffing behavior considered in this study such as puff volumes (55, 65, and 75 ml), puff duration (4 s), and puff interval (30 s) were recommended by methods such as CORESTA, previous empirical studies, and/or documented in cytotoxic investigations for EVPs-associated research applications (26, 42, 43, 47, 48, 50, 62–65). Though the pod-style EVP user has no control over power settings, one of the goals of this study was to assess the influence of puff volumes (55, 65, and 75 ml) by heating the reference e-liquid at power settings (6.5 and 7.5 W) on PSD and respiratory deposition using the pod-style EVP. MMADs for all the puff profiles with the reference e-liquid were ~1 μm, which is consistent with the finding presented by Lechasseur et al. (40). In their study, the authors noted e-liquids with a higher VG component with a PG:VG ratio of 30:70 resulted in the emission of larger particles than a 70:30 PG:VG ratio. Our observation is that with 55- and 65-ml puff volumes, increasing the power delivery could generate larger particles, possibly because of higher particle density by aerosolizing more e-liquid material, is consistent with the earlier studies that evaluated an EVP design prior to a pod-style design (40, 66). Despite not statistically significant, MMAD with a 75-ml puff volume was 1.02 μm at 7.5 W compared to 1.10 μm at 6.5 W, possibly because of the emission of smaller-sized particles at a higher puff volume, which lowered the size distribution at a higher power. Mikheev et al. (41) documented a reduction in particle size at a higher puff flow rate beyond 25 ml/s because of evaporation, presumably a result of higher dilution while sampling aerosols. Compared to Mikheev's study, we sampled the three puffs of reference e-liquid EVP aerosols with puff volumes of 55, 65, and 75 ml within 4 s, each 30 s apart. This led us to sample a total of 165, 195, or 225 ml for every trial at a constant sampling flow rate of two LPMs to evaluate MMAD using MiniMOUDI™. Additionally, at given experimental parameters, we did not measure significant differences in MMADs because of evaporation with different puff profiles aerosolizing the reference e-liquid at 7.5 W. At higher power settings (7.5 W), our measurement at a higher puff volume (75 > 65 > 55 ml) caused the particle size to decrease (MMAD (μm) 1.02 <1.13 <1.23), which was consistent with the findings from Li et al. (48). Higher puff volumes, possibly providing less time for particle collision or coagulation, coupled with a higher power increased the heating of the e-liquid and decreased the aerosol size emitted.

Complexity in determining the respiratory deposition of EVP aerosols based on their size distribution has been addressed in previous studies (29–34, 36). Raabe et al. (67) concluded that the highest pulmonary deposition fraction was for particles with MMAD smaller than 2 μm, which would deposit in the lower respiratory tract. At particle sizes ranging from 0.2 to 1 μm, higher deposition fractions in the lower lobes compared to the upper lobes were also documented, as observed in our results (53, 68–71). Lechasseur et al. (40) presented the conditions that an increase in the particle size aerosolized by heating e-liquids with a PG–VG ratio of 30:70 at higher power settings led to a reduction in pulmonary deposition. Dosimetry results presented in this study for various puff profiles are consistent with these authors' observations. Other than the highest MMAD (1.23 μm), 55-ml puff volume at 7.5 W, all puff profiles resulted in the highest pulmonary deposition. Compared with other puff profiles, this puff profile resulted in 41% of mass deposition in the head, which is comparable with 40% in pulmonary regions. The literature has evidentially presented that particles smaller than 1 μm are not only known to result in deep lung deposition but also are able to be exhaled with a greater chance (29, 30). Smaller-sized particles could serve as a potential secondhand exposure on exhalation to nearby people, especially in occupational settings such as vape shops. More than 64% of the aerosols emitted from all the puff profiles studied were estimated to be exhaled. The direct measurement of exhaled aerosols should be conducted to determine the correct PSD of secondhand exposure conditions as primary aerosols inhaled differ from the aerosol exhaled by an EVP user. The results of the percentage of exhaled aerosol presented here indicated that pod-style EVP aerosols can potentially serve as a secondhand exposure for employees in occupational settings (e.g., vape shops, bars, and restaurants) that allow to use in indoors.

### Commercially Available JUUL^®^ e-Liquids

After FDA restrictions on the sale of flavored prefilled pods in 2020, other fourth generation EVP devices became available and popular in the market that can aerosolize various flavored e-liquids, other than the prefilled JUUL^®^ brand pods flavored with Menthol and Tobacco (45, 72, 73). Furthermore, nicotine salt mixed with a custom-made e-liquid bulk material to be used in refillable pod-style devices could provide all the flavors reported in a previous study (74). Additionally, this ban only applied to the flavored cartridges for use in pod-style EVP devices and does not apply to manufacturers of any other flavored e-liquid that is attached to the mouthpiece of JUUL^®^ and other brand pod-style EVP devices. It is noteworthy that other manufacturers have developed the flavored e-liquid pods that are attached to the mouthpiece of JUUL^®^ and other brand pod-style EVPs (75). However, adequate research on the physical characteristics of the particles, such as PSD emitted from the pod-style EVPs, has until now been lacking and this information is necessary to understand regional lung depositions. We studied commercial JUUL^®^ pods, either currently available or not, to have an idea about PSD and lung deposition by aerosolizing the prefilled flavored JUUL^®^ e-liquids.

Menthol-containing JUUL^®^ flavors resulted in lower MMADs [5%: 0.92 μm (the lowest); 3%: 1.11 μm] compared with the other studied JUUL^®^ pods. This result was consistent with a previous observation by Lechasseur et al. (40) evaluating the effect of menthol on e-liquid with nicotine in 30:70 PG–VG. Lamb et al. (76) studied the cytotoxicity of currently available JUUL^®^ flavor aerosols: Menthol and Virginia Tobacco. The authors indicated that an exposure to Menthol-flavored JUUL^®^ pods causes considerable mitochondrial dysfunction in lung epithelial cells compared with Virginia Tobacco. Depending on MMAD and GSD, of all the studied JUUL^®^ pods, Menthol 5% resulted in the highest pulmonary (42%) and TB (23%) depositions and the lowest head (35%) and total respiratory (28%) depositions. An inversely related percentage of exhaled EVP aerosols (72%), a secondhand exposure estimate, was the highest for Menthol 5%. A consideration of the exhalation of smaller-sized particles was addressed in this study by presenting the estimates of exhaled fraction as a surrogate for potential secondhand exposure conditions. Inversely related to total respiratory deposition, an average of 58%, EVP aerosols ranged between 52% (the lowest with Mango 5% nicotine) and 72% (the highest with Menthol 5% nicotine). Other than Menthol 5%, the rest of the studied JUUL pods resulted in larger sized particles with MMAD >1 μm and wider size distributions with GSD > 2. Lechasseur et al. (40) indicated larger sized particles deposited at regions other than the pulmonary region. In our dosimetry evaluations, all the JUUL pods other than Menthol 5% resulted in a higher deposition in the head (43–52%) rather than in the pulmonary region (30–37%). Some studies have observed an association between an EVP user and respiratory symptoms among adolescents (77, 78), a reduced pulmonary immune function (65, 79), and an increased risk of mood and attention symptoms (80) as well as potential long-term effects on brain development for cognitive behavior (81, 82). Pearce et al. (83) characterized the aerosolized JUUL^®^ Fruit Medley flavored e-liquid and documented a reduced cellular metabolic activity in a dose-dependent manner. Stefaniak et al. (16) reviewed the toxicology of flavorings used in e-liquids. Apart from the existing cytotoxicity studies, our findings of pulmonary deposition could help explain the development or exacerbation of respiratory and systemic toxicity from the use of aerosolized JUUL^®^ e-liquids. Though some JUUL^®^ flavors are restricted from sale in the USA, the currently available Menthol and Virginia Tobacco JUUL^®^ flavors can potentially lead to considerable health damages (76). Additionally, the aerosolization of flavored e-liquids using refillable blank pod-style EVPs can be as harmful as JUUL^®^ flavors (75).

## Challenges and Limitations

For the fourth generation EVPs, a reference model is not available like those of the second and third generation EVPs (84). Study results may deviate using the different types of blank pod-style devices than those used in this study. The refillable pods from other brands may not have identical characteristics, such as coil material and surface area, compared to the JUUL^®^ brand. However, we used blank pods comparable to JUUL^®^ brand pods in reservoir size and electric capability to aerosolize the reference e-liquid.

Without standardized experimental protocols, parameters included in any study could be a source of limitations that can cause a lack of reproducibility and comparability among different studies. The ECAG+ used in our study was based on the principles of positive pressure to generate aerosols rather than the negative pressure used by humans. We are unaware of any study that has compared PSDs generated by positive and negative pressure devices for the same e-liquid so the influence of this experimental parameter on results is unknown at this time. Depending on these experimental and sampling parameters, the size distribution of EVP aerosols deviates because of hygroscopic growth and evaporation in the human lung environment. These deviations could be impacted by various experimental parameters such as the composition of e-liquids, puff volume, and power settings (32, 36, 40, 85, 86). These factors play a significant role in the determination of PSD and therefore the regional deposition of the aerosols in the respiratory tract. Influences of variables such as the puff volume and power setting are more relatable to JUUL^®^ and other pod-style devices, which are flow-activated, low-powered EVPs.

Even with all variables held constant, Protano et al. (87) demonstrated significant variations in puff-to-puff aerosol generation within a single EVP device. Mass measurement with JUUL^®^ EVPs was challenging because of a variability in aerosol generation, as documented in a previous study (7). To better understand PSD, two different power settings were considered in this study, however, to avoid damages to the physical integrity of a pod-style EVP, we could not report the temperatures of e-liquid while puffing at 6.5 and 7.5 W. As JUUL^®^ devices are flow-activated EVP design types, it was difficult to determine estimates for power settings and thereby the temperature of e-liquid while puffing at the given puff profiles among EVP users in the real-world scenarios. Therefore, for JUUL^®^ pods, the results of this study are limited to one fixed puff topography, which was a 4-s puff, 30-s interval, and 65-ml puff volume by aerosolizing e-liquid at 7.5 W.

As the focus of our study was to evaluate the influential puffing parameters affecting PSD using pod-style EVPs, we considered using a puff profile that included those influential parameters related to determining PSD and ultimately respiratory deposition. The influential parameter conditions (e.g., puff volumes, puff flow rates, electrical settings, and devices) considered herein might be different from those found in real-world scenarios or used by other research studies. Hence, PSD depending on these parameters might be affected and this would impact aerosol respiratory deposition estimates. Additionally, the estimated respiratory deposition fractions using the MPPD software were not modified for hygroscopic growth and evaporation according to the human lung environment. The dosimetry analysis did not consider the clearance mechanisms that may impact total respiratory deposition and exhaled aerosol estimates. This report, being unique in addressing the PSD of pod-style EVPs, should stimulate additional experiments regarding different puff topographies for JUUL^®^ pods in the future and focus on characterizing the chemical content of the vapor exhaled by an individual using a pod-style EVP.

## Summary

After FDA restrictions on prefilled EVP flavor pods other than Menthol and Tobacco, refillable pods have been more popular to aerosolize various flavors of homemade or commercially available e-liquids. Although toxicological studies have reported evidence of deleterious health effects on heating e-liquids in pod-style EVP design—either prefilled in JUUL^®^ or simulated JUUL^®^-like e-liquids, the PSD of emitted EVPs aerosol as a significant determinant for their regional respiratory depositions has not been addressed adequately (28, 65). This study evaluated respiratory tract depositions as a function of the MMAD and GSD of particles emitted by heating the simulated reference e-liquid that mimicked JUUL^®^ e-liquid at 6.5 and 7.5 W with the three puff volumes (55, 65, and 75 ml). The higher 7.5 W power setting was an influential factor that significantly impacted MMADs at 55- (1.23 μm) and 75-ml (1.02 μm) puff volumes. In general, for all puff profiles with the reference e-liquid, the dosimetry analysis predicted that 40–43% of total respiratory depositions of particles were in the pulmonary regions where toxicological implications have been reported. With wider size distributions for JUUL^®^ e-liquids (GSD: 1.69–2.75), dosimetry modeling predicted comparable particle depositions in the head (35–52%) and in the pulmonary regions (30–42%). Irrespective of statistical differences in their size distribution, the emitted aerosols from heating the reference e-liquid or JUUL^®^ e-liquids are predominantly (60%) deposited in the lower lobes (Right 30% and Left 30%) of the lungs. Inversely related to the estimated total respiratory deposition, more than 52% of the aerosols were exhaled, which could potentially serve as secondhand exposure conditions at workplace and hence needs to be assessed in indoor environments.

## Data Availability Statement

The original contributions presented in the study are included in the article/Supplementary Material, further inquiries can be directed to the corresponding author/s.

## Author Contributions

All authors listed have made a substantial, direct, and intellectual contribution to the work and approved it for publication.

## Author Disclaimer

The findings and conclusions in this report are those of the authors and do not necessarily represent the official position of the National Institute for Occupational Safety and Health, Centers for Disease Control and Prevention. Mention of any company or product does not constitute endorsement by the US Government, National Institute for Occupational Safety and Health, or Centers for Disease Control and Prevention. This work was supported by NIOSH intramural research funds.

## Conflict of Interest

The authors declare that the research was conducted in the absence of any commercial or financial relationships that could be construed as a potential conflict of interest.

## Publisher's Note

All claims expressed in this article are solely those of the authors and do not necessarily represent those of their affiliated organizations, or those of the publisher, the editors and the reviewers. Any product that may be evaluated in this article, or claim that may be made by its manufacturer, is not guaranteed or endorsed by the publisher.

## Supplementary Material

Supplementary Table 1 Mass median aerodynamic diameter (MMAD) and geometric SD (GSD) for individual trials for puff profiles of the reference e-liquid.
