# Supplementary material for: Effect of Puffing Behavior on Particle Size Distributions and Respiratory Depositions From Pod-Style Electronic Cigarette, or Vaping, Products
Source: Front Public Health. 2021 Dec 1;9:750402. doi: 10.3389/fpubh.2021.750402 (PMC8671759; doi:10.3389/fpubh.2021.750402)
Supplement: Supplementary Table 1 — Mass median aerodynamic diameter (MMAD) and geometric SD (GSD) for individual trials for puff profiles of the reference e-liquid. [file Table_1.pdf]

Supplemental Material

Effect of Puffing Behavior on Particle Size Distributions and Respiratory Depositions from Pod-style Electronic Cigarette, or Vaping, Products

Anand Ranpara*, Aleksandr B. Stefaniak, Elizabeth Fernandez, Ryan F. LeBouf

Respiratory Health Division, National Institute for Occupational Safety and Health,
1095 Willowdale Road, Morgantown, WV, United States

*** Correspondence: Ryan F. LeBouf, RLeBouf@cdc.gov**

Keywords: E-cigarette, Pod-style E-cigarette, JUUL®, Particle size distributions, Respiratory deposition, Secondhand exposure estimates

Supplemental material

Table S1: Mass median aerodynamic diameter (MMAD) and geometric standard deviation (GSD) for individual trials for puff profiles of reference e-liquid.

Puff profile	Power (W)	Puff Volume (mL)	Note	MMAD (µm)	GSD
1	6.5	55		1.11	1.63
1	6.5	55		1.09	1.59
1	6.5	55		1.11	1.59
2	6.5	65		1.08	1.58
2	6.5	65		1.14	1.60
2	6.5	65		1.10	1.58
3	6.5	75		1.16	1.62
3	6.5	75		1.10	1.58
3	6.5	75		1.03	1.49
4	7.5	55		1.21	1.69
4	7.5	55		1.25	1.71
4	7.5	55		1.23	1.71
4*	7.5	55	Evaporative mass loss	1.20	1.67
4*	7.5	55	Evaporative mass loss	1.25	1.71
4*	7.5	55	Evaporative mass loss	1.24	1.71
5	7.5	65		1.19	1.65
5	7.5	65		1.12	1.63
5	7.5	65		1.08	1.62
5*	7.5	65	Evaporative mass loss	1.20	1.65
5*	7.5	65	Evaporative mass loss	1.05	1.54
5*	7.5	65	Evaporative mass loss	1.06	1.55
6	7.5	75		0.97	1.37
6	7.5	75		1.11	1.58
6	7.5	75		0.97	1.40
6*	7.5	75	Evaporative mass loss	0.97	1.38
6*	7.5	75	Evaporative mass loss	1.08	1.55
6*	7.5	75	Evaporative mass loss	0.97	1.35
